# Balancing continuity of care and liability concerns: One community pharmacy experience of a person with type 1 diabetes using a do-it-yourself automated insulin delivery system

**DOI:** 10.1177/17151635251373080

**Published:** 2025-10-28

**Authors:** Youssef A. Elezzabi

**Affiliations:** Faculty of Pharmacy and Pharmaceutical Sciences, College of Health Sciences, University of Alberta, Edmonton, AB

## Introduction

Individuals living with type 1 diabetes (T1D) require multi-daily insulin administration and glucose monitoring to maintain health and reduce the risk of diabetes-associated complications. Maintaining glucose levels within a target range set with the individual’s diabetes team and in accordance with clinical guidelines seeks to minimize the incidence of hyper- and hypoglycemia and the associated short-term and long-term risks thereof. With carbohydrate quantity, macronutrient composition of food, physical activity, illness, hormone fluctuations, stress, and numerous other factors that unpredictably affect blood glucose, managing T1D can burden patients.

Seeking to reduce this burden and provide more effective management,^1,2^ hybrid closed-loop automated insulin delivery (AID) systems, also informally termed the “artificial pancreas” and “looping”, have increased in prevalence since being developed by the open-source community in 2013 in a do-it-yourself (DIY) model. This model requires patients to build mobile applications based on free and publicly available code due to legislative requirements that would prohibit the official marketing of such applications in a built form unless approved by regulatory agencies such as Health Canada.^3^ These systems pair a continuous glucose monitor (CGM) with an insulin pump to administer varying dosages of insulin or halt its administration automatically in response to glucose levels and rate of change, reducing patient burden of intervention to only entering carbohydrates while aiming to improve clinical indicators (decreased HbA1C and increased time in range).^4^ Looping turns T1D from a reactive to a proactive condition. Yet, it is not a functional cure, primarily due to the 5- to 15-minute CGM delay from the sampling of interstitial glucose and the pharmacokinetic absorption delay of subcutaneously infused insulin.^1^

Current approved closed-loop systems on the Canadian market include systems made by Medtronic, Tandem, and Insulet.^3,5^ However, the prevalence of DIY AID systems, which involves patients building a specific phone application based on open-source code, has increased over the years because the flexibility of CGM and pump choices is unparalleled, and there is an absence of regulatory delays and fees that delay patient access. As well, DIY AID systems offer greater customizability than marketed systems. How, then, can pharmacists provide patient-centred care while navigating the legal liabilities and lack of training around these unapproved systems, considered “Software as a Medical Device” under Health Canada regulations?^6^ While some literature has explored this topic from physician and patient perspectives,^3,6,7^ limited literature from a pharmacy practice-specific perspective exists.^8,9^ Adoption and dispensing of medical devices such as CGM systems have increased over the years, and pharmacists will undoubtedly see more patients using AID systems, both DIY and official. This publication analyzes and comments on a case in which a patient expressed concern for the care they received in a community pharmacy, specifically regarding the refusal to fill an insulin prescription that they attributed to the pharmacist’s liability concerns, and provides recommendations on how we, as a profession, can adopt this technology while continuing to provide patient-centred care and mitigate liability concerns.

## Description of case

The patient currently lives with T1D and uses DIY AID with the Dexcom G6 and Omnipod DASH, with an HbA1C <7% and time in range >80%.

I interviewed this patient for one of my PharmD program assignments. Their story intrigued me, and I asked the patient for a second interview to collect further details and consent to publish their story.

Having been on the Alberta Insulin Pump Therapy Program (IPTP) waitlist for 4 years, the patient was accepted at 23 years of age. The patient was using the FreeStyle Libre 1 flash glucose monitor paired with the MiaoMiao transmitter to allow for continuous glucose monitoring and began using the Omnipod EROS insulin pump.

Approximately 1 week after initiating pump therapy, and with an endocrinologist supportive of DIY AID, the patient obtained an insulin lispro prescription and went to their regular community pharmacist that they had known since childhood, voluntarily and enthusiastically disclosing to their pharmacist that they would be looping. The pharmacist then refused to fill the prescription, citing liability concerns because this was a perceived unauthorized use of the prescription. The pharmacist instructed the patient to obtain a new prescription to ensure that the prescribing physician approved of looping. Additionally, according to the patient, the pharmacist flagged the patient’s file and placed a note in it and, furthermore, contacted the patient’s diabetes clinic, informing them that the patient was looping, which the patient perceived as the pharmacist reporting them.

Unaware that they could have presented the initial prescription to a different pharmacy to be filled, the patient instead visited their family physician and obtained a new prescription. The patient returned to the same pharmacy a few days later with the new prescription and stated that they would not be looping, hoping to avoid a repeat encounter of prior events. The patient expressed that they felt the pharmacist’s refusal to fill caused them to exhaust their insulin supply except for what remained in their pump, placing them at risk of acute complications such as diabetic ketoacidosis. Additionally, the patient did not feel a sense of safety seeking care from that community pharmacist and felt that they needed to withhold information about their health and treatment regimen to continue receiving their prescriptions.

## Discussion

Unfortunately, I did not have a chance to interview the community pharmacist to seek their perspective. The patient did not disclose the name of the community pharmacist and pharmacy, nor did I ask. The patient consented to the publication of their lived experience but wished to remain anonymous and non-identifiable. As such, contacting the community pharmacist would have compromised the patient’s confidential disclosure and may have caused the community pharmacist to feel that the patient reported them or that they, as the community pharmacist, were being investigated. Accordingly, only the patient’s lived experience was sought for this reason. Although this is a limitation to a fully impartial analysis of the event, I assume that the community pharmacist did not intend to harm the patient, but harm in the relationship with the patient resulted, possibly due to lack of education that resulted in the refusal to fill.

Initially enthusiastic to loop after having waited a substantial amount of time for IPTP coverage, the patient felt that this experience violated their trust and confidence in pharmacists. When the patient ran out of insulin, they skipped educational obligations to avert a medical emergency by promptly obtaining another prescription from their family physician. Overall, the patient felt that they were navigating an unsupportive health system and had to withhold that they were looping in order to continue to receive life-sustaining medication. As a result, they no longer voluntarily disclose they are looping to pharmacists, fearing a similar experience.

The patient noted that chastising is an improper strategy for pharmacists. Patients may cope with chronic conditions in unconventional ways that are not officially approved, but that does not mean they are wrong to do so. Pharmacists need to meet the patient where they are at, or the patient will simply not disclose their management strategies. Thus, the patient care record will be incomplete, hindering optimal care and coordination thereof.

Although pharmacists are not required to provide official guidance on DIY AID settings due to the legal liabilities, they can still do much to provide patient-centred care and support the autonomy of looping patients. Pharmacists likely do not receive formal training on DIY AID systems because they are unapproved; however, self-driven education through open-source documentation, online videos, scientific literature, and patient interactions to supplement this possible lack of training is essential to providing patient-centred care. Lack of knowledge on DIY AID was likely a major factor in the decision to refuse to fill, with the pharmacist likely fearing liability should the patient experience an adverse drug event.

Furthermore, if pharmacists simply support patient autonomy and create safe spaces for patients to be open, this is a form of harm reduction, given that the patient assumes responsibility for any harms in using unapproved AID systems.^6^ Most important, knowledge that a patient is looping should never compromise patient care, and prescriptions—especially those for a life-sustaining medication like insulin—should not be denied.

## Conclusion

Recommendations for pharmacy practice were co-developed with the patient. Pharmacists play a crucial role in diabetes care and need to create safe spaces for patients who chose to use DIY AID systems. At a minimum, the patient’s decision to loop should not revoke their eligibility to receive life-sustaining medication. Rather, a harm reduction approach must be taken. While there are liability concerns for pharmacists around supporting looping, pharmacists can still learn about looping and respect patient autonomy. This guidance is especially relevant to pharmacists specializing in diabetes, such as current and prospective certified diabetes educators. Given that clinician lack of knowledge about DIY AID was likely a root cause in the patient’s experience, further research into the current level of education of DIY AID and AID systems in general across Canadian pharmacy schools and continuing education programs offered through the various pharmacist associations is needed to ensure pharmacists are well-trained in this area. ■
